# Key issues for implementation of Genomics in Healthcare: a Policy Brief

**DOI:** 10.1093/eurpub/ckac131.022

**Published:** 2022-10-25

**Authors:** A Costa, A Merchant, MF Lopes, M Konopko, ML Cardoso, X Sitjà, M Bourbon, S Scollen, A Vicente

**Affiliations:** 1 Department of Health Promotion and NCD Prevention, National Institute of Health Doutor Ricardo Jorge, Lisbon, Portugal; 2 Institute of Social and Political Sciences, University of Lisbon, Lisbon, Portugal; 3 ELIXIR Hub, Wellcome Genome Campus, Cambridge, UK; 4 BioISI-Biosystems & Integrative Sciences Institute, Faculty of Sciences, University of Lisbon, Lisbon, Portugal

## Abstract

**Issue/problem:**

Healthcare (HC) can significantly benefit from genomic information for earlier, accurate diagnosis, effective personalized treatment with less adverse events, and accurate profiling of individuals for disease prevention. However, European countries are currently at variable maturity stages regarding the implementation of genomic medicine (GM) in healthcare, hindering the equitable delivery of personalized medicine to citizens across borders.

**Description of the problem:**

The European 1+Million Genomes Initiative (1+MG) aims to provide cross-border access to quality genomic information and related clinical data, to advance data-driven research and HC solutions to benefit citizens. This initiative is encouraging countries to develop national GM strategies, but guidance for successful implementation is needed. In this context, the Beyond 1 Million Genomes, a supporting action to the 1+MG initiative, organized three Country Exchange Visits (CEV) to discuss critical issues, share experiences and best practices, for the implementation of sustainable GM strategies in healthcare.

**Results:**

The United Kingdom, Estonia and Finland, which have advanced GM programs, hosted CEV describing progress and lessons learnt. Representatives of 1+MG signatory countries participated in these events and were able to present country level progress. The resulting Policy Brief (PB) captures key issues discussed at the CEVs, with real-life examples, and proposes policy recommendations for the successful implementation of GM in European healthcare systems.

**Lessons:**

Sustainable GM implementation in HC systems requires: 1) Patient and citizens trust and engagement; 2) Sustainable infrastructure and data regulation, with solid ethical and legal frameworks; 3) Capacity building of healthcare professionals; 4) A strong ecosystem involving all stakeholders, and encouraging synergies between healthcare, research and industry to promote continuous innovation.

**Key messages:**

• The implementation of GM in healthcare will take countries further towards making personalized medicine a reality, with remarkable health and socioeconomic benefits for patients and healthcare systems.

• Promoting cooperation, capacity building and sharing of best practices is crucial to reduce asymmetries between countries, which constrains effective and equitable cross-border personalized medicine.

